# Prediction of MAFLD and NAFLD using different screening indexes: A cross-sectional study in U.S. adults

**DOI:** 10.3389/fendo.2023.1083032

**Published:** 2023-01-19

**Authors:** Hongye Peng, Liang Pan, Simiao Ran, Miyuan Wang, Shuxia Huang, Mo Zhao, Zhengmin Cao, Ziang Yao, Lei Xu, Qing Yang, Wenliang Lv

**Affiliations:** ^1^ Department of Infection, Guang’anmen Hospital, China Academy of Chinese Medical Sciences, Beijing, China; ^2^ Phase 1 Clinical Trial Center, Deyang People’s Hospital, Sichuan, China; ^3^ Department of Gastroenterology, HuangGang Hospital of Traditional Chinese Medicine (TCM) Affiliated to Hubei University of Chinese Medicine, Huanggang, Hubei, China; ^4^ School of Public Health, Tongji Medical College, Huazhong University of Science and Technology, Wuhan, Hubei, China; ^5^ School of Foreign Languages and Culture, Nanchang University, Nanchang, Jiangxi, China

**Keywords:** metabolic dysfunction-associated fatty liver disease, waist circumference, lipid accumulation product, triglycerideglucose index, BMI, TyG-WC, WHtR, TyG-WHtR

## Abstract

**Introduction:**

Metabolic dysfunction-associated fatty liver disease (MAFLD), formerly known as non-alcoholic fatty liver disease (NAFLD), has become the most common chronic liver disease worldwide. We aimed to explore the gender-related association between nine indexes (BMI/WC/VAI/LAP/WHtR/TyG/TyG-BMI/TyG-WC/TyG-WHtR) and MAFLD/NAFLD and examine their diagnostic utility for these conditions.

**Methods:**

Eligible participants were screened from the 2017-2018 cycle data of National Health and Nutrition Examination Survey (NHANES). Logistic regression and receiver operating characteristic (ROC) curve were used to assess the predictive performance of 9 indexes for MAFLD/NAFLD.

**Results:**

Among the 809 eligible individuals, 478 had MAFLD and 499 had NAFLD. After adjusting for gender, age, ethnicity, FIPR and education level, positive associations with the risk of MAFLD/NAFLD were found for all the nine indexes. For female, TyG-WHtR presented the best performance in identifying MAFLD/NAFLD, with AUC of 0.845 (95% CI = 0.806-0.879) and 0.831 (95% CI = 0.791-0.867) respectively. For male, TyG-WC presented the best performance in identifying MAFLD/NAFLD, with AUC of 0.900 (95% CI = 0.867-0.927) and 0.855 (95% CI = 0.817-0.888) respectively.

**Conclusion:**

BMI/WC/VAI/LAP/WHtR/TyG/TyG-BMI/TyG-WC/TyG-WHtR are important indexes to identify the risk of MAFLD and NAFLD.

## 1 Introduction

Non-alcoholic fatty liver disease (NAFLD) is a syndrome including non-alcoholic fatty liver, non-alcoholic steatohepatitis, associated cirrhosis, liver cancer, and other diseases. It is defined by excessive fat accumulation in hepatocytes that is not caused by alcohol or other clear liver injury. NAFLD is the most common chronic liver disease in the world today, affecting the health of 25.24% adults (1). Previous studies have confirmed that NAFLD is closely associated with several metabolic diseases, such as hyperlipidemia, diabetes (2), and hypertension (3).NAFLD has been commonly linked to the metabolic syndrome (MetS). The 2020 International Expert Consensus recommended renaming NAFLD as metabolic dysfunction-associated fatty liver disease (MAFLD) to better meet clinical and research needs due to the rising prevalence of NAFLD, the improved understanding of its pathogenesis, and the drawbacks and shortcomings of previous exclusionary diagnoses (4). Although some existing studies suggest that MAFLD may be more advantageous in identifying advanced fibrosis and metabolic abnormalities, there still is limited evidence and not much research on MAFLD (5).

Patients with fatty livers have the risk of not only developing in cirrhosis and liver cancer, but also developing diabetes, cardiovascular disease, and kidney disease (6), which seriously affect their life quality and health. According to Younossi et al., NAFLD is anticipated to affect over 64 million people in the United States, with direct medical costs of about $103 billion annually ($1,613 for each patient) (7). Additionally, the prevalence of adult obesity, diabetes, and aging will all contribute to an increase in NAFLD-related liver disease and mortality. More than 800,000 liver deaths are projected between 2015 and 2030 (8). Therefore, early diagnosis and identification of fatty liver disease is critical in safeguarding the health of the population and reducing the financial burden of national health. Pathological biopsy, a gold standard for diagnosing fatty liver disease, is expensive, invasive, and accompanied by postoperative complications (9). Exploring easy-to-use, practical, and reliable predictors of fatty liver disease is clinically significant and valuable.

Obesity is one of the common causes of hepatic steatosis and is closely linked to insulin resistance (IR). However, increasing studies suggest that adipose tissue has a variety of functions and some adipose tissue is harmless to the body, such as brown fat (10). In addition, the distribution site of adipose tissue is also closely related to health status (11). We cannot simply assume that high body weight and excessive fat accumulation indicate poor health status since there are phenotypes of metabolically healthy obese (MHO) and metabolically unhealthy non-obese (MUNO) (12, 13). Visceral adiposity and lipid accumulation may, to some extent, assess more accurately the role of adipose tissue in the physiopathological processes as well as its value in predicting disease risk. Visceral adiposity index (VAI), proposed by Amato et al. (14), is a novel body fat index integrating waist circumference, body mass index (BMI), triglycerides (TG), and high-density lipoprotein cholesterol (HDL-C) and is considered a reliable predictor of visceral adiposity. The relationship between VAI and metabolism-associated diseases is also widely investigated recently. A 4-year prospective cohort study suggested that VAI level was an independent risk factor for NAFLD, and there was a dose-response relationship between them (15). Lipid accumulation product (LAP), an easily accessible index consisting of waist circumference and triglycerides, may better reflect the extent of lipid accumulation compared to central obesity alone (16). Dai et al. confirmed that LAP was highly linked to the incidence and severity of NAFLD and a reliable predictor of NAFLD risk in Chinese adults (17). Triglyceride-glucose (TyG) index, a reliable surrogate for IR assessment, is closely associated with cardiovascular disease (18), diabetes mellitus (19), diabetic nephropathy (20) and various diseases. TyGis found to be important in identifying individuals at risk for NAFLD and assessing the progression of liver fibrosis (21, 22). More studies suggest that TyG-BMI, TyG-WC and TyG-WHtR are reliable indicators for NAFLD (23–25). BMI is widely used for obesity measurement, while waist circumference (WC) and waist-to-height ratio (WHtR) are important indicators for central obesity assessment. However, there is little research on the differences among BMI, WC, VAI, LAP, WHtR, TyG, TyG-BMI, TyG-WC and TyG-WHtR in predicting the risk of MAFLD/NAFLD.

This study intends to explore the differences among those indexes in predicting the risk of MAFLD/NAFLD based on the data of US adults in the 2017-2018 cycle from National Health and Nutrition Examination Survey (NHANES), aiming to provide a reliable reference for early detecting and identifying indicators of MAFLD/NAFLD.

## 2 Materials and methods

### 2.1 Study design and participants

**Figure 1:**
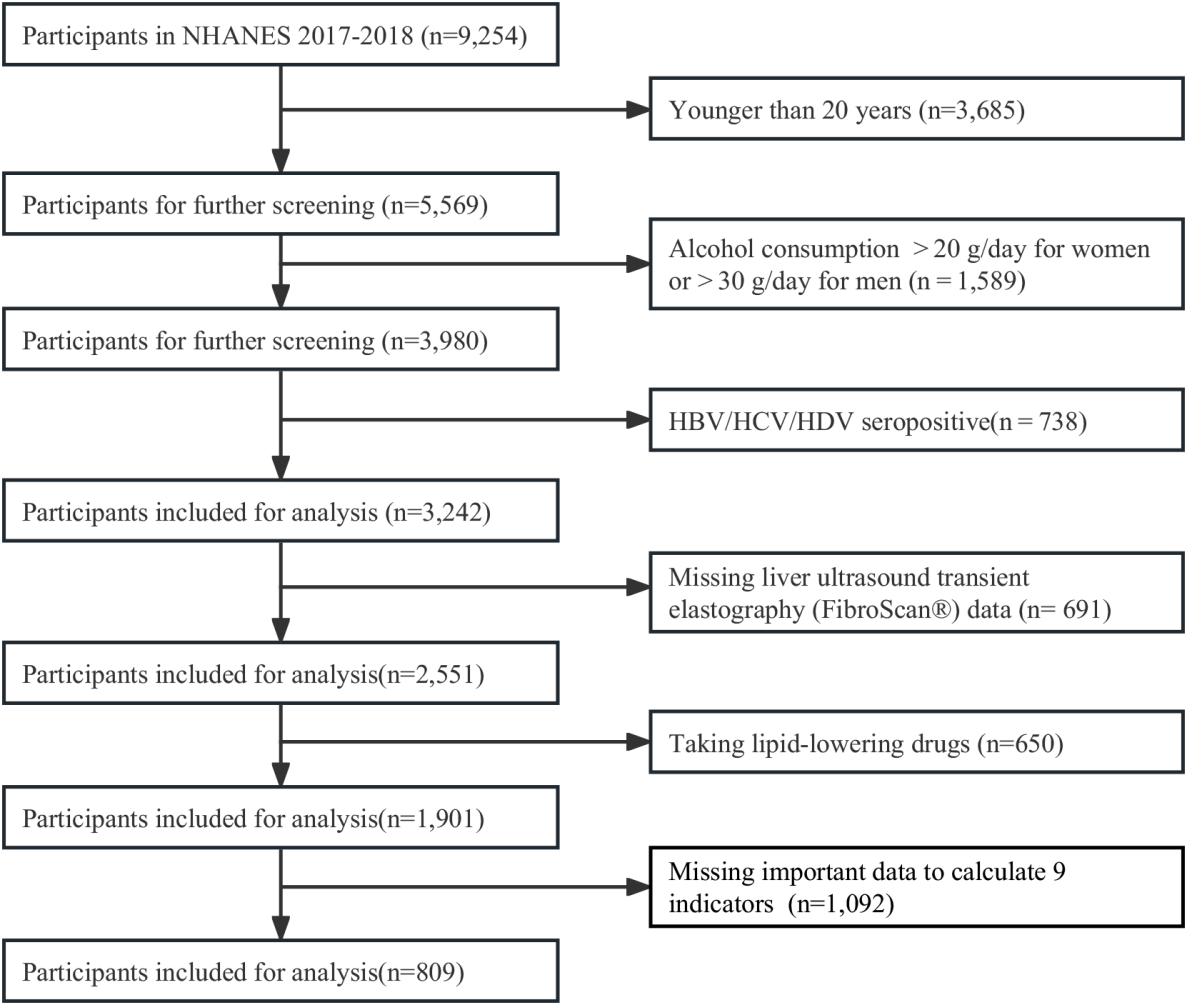
Flow chart for the selection of participants in the cross-sectional study.

All individuals aged ≥ 20 years from the cycle 2017 to 2018 of the NHANES in the United States were screened in this study. Profiles of the NHANES were described in previous study (26). The NHANES gathered a representative sample from the non-institutionalized U.S. population using a complicated, multi-stage, and probability sampling strategy. All data were collected with household interviews, mobile physical examinations, and laboratory tests. The participant screening flow chart was displayed in **Figure 1**. From all 9,254 individuals, we excluded participants aged< 20 years (*n* =3,685), drinking heavily (*n*=1,589), positive serology for hepatitis B, C and D (*n*=738), missing data of liver ultrasound transient elastography (FibroScan^®^) (*n*= 691), taking lipid-lowering drugs (*n*=650), and missing important data to calculate 9 indicators (*n* = 1,092). Finally, 809 participants were included for analysis. The Research Ethics Review Board of the National Center for Health Statistics examined and approved the NHANES protocol. Each participant completed a written statement of informed consent.

### 2.2 Definition of MAFLD and NAFLD

MAFLD was defined by presence of hepatic steatosis (HS) on ultrasound and meeting at least one of three conditions: overweight/obesity, presence of T2DM, or presence of metabolic disorder (27). Lean/normal-weight individuals with HS but no T2DM were considered to have a metabolic disorder if two or more of the following metabolic risk abnormalities were present: 1) WC ≥102cm in males or 88 cm in females, 2) blood pressure ≥130/85mmHg or specific medications, 3) serum TG ≥1.70mmol/L or specific medications, 4) HDL-C< 1.0mmol/L for males and < 1.3mmol/L for females, 5) prediabetes (a fasting glucose level between 5.6 and 6.9mmol/L, or a 2-hour post-load glucose level between 7.8 and 11.0mmol/L or an hemoglobin A1c (HbA1c) level between 5.7% and 6.4%), 6) a HOMA-IR score ≥ 2.5 and 7) a plasma C-reactive protein level > 2mg/L.

NAFLD was defined by presence of HS on ultrasound, excluding heavy drinking individuals (those consuming alcohol > 20 g/day for females or > 30 g/day for males) and other competing etiology for HS (those with hepatitis B/C/D positive serology). Considering that transient elastography (FibroScan^®^, TE) with controlled attenuation parameters (CAP) presented good accuracy in determining the level of hepatic steatosis (28), HS was diagnosed by FibroScan with CAP values ≥238 dB/m (29).

### 2.3 Nine indirect indexes and laboratory measurement

All participants were interviewed at home and physically examined at a mobile examination center (MEC). They were also required to fast at least nine hours before blood sampling. Height and weight were measured at the MEC following protocol and then used to calculate BMI, rounding to one decimal place. At the end of a normal exhale and while standing naturally with the legs spread out approximately 25-30 cm apart, WC was measured using an inelastic ruler with a minimum scale of one millimeter. The ruler was placed at the midpoint of the connecting line between the upper edge of the top of the iliac crest and the lower edge of the 12th rib (the narrowest part of the waist) and circled horizontally the abdomen, and readings were rounded to 0.1cm (30). After resting for at least 5 minutes, participants were measured with blood pressure using a standardized mercury sphygmomanometer in a sitting position.

Laboratory methods for measuring lipid profile, HbA1c, glucose, insulin, and plasma C-reactive protein level were described by CDC (31).

Alcohol consumption was calculated with self-reported information on drinking status within the last year. The consumed alcohol was reported in standard drinks and converted to grams using a multiplication factor of 14.

Indexes for assessment were calculated by using the following formulas (14, 16, 32):

BMI=weight (kg)/height^2^ (m);
VAI=WC (cm)/(39.68 + 1.88 x BMI (kg/m^2^)) x TG (mmol/L)/1.03 x 1.31/HDL-C (mmol/L) for males
VAI=WC (cm)/(36.58 + 1.89 x BMI (kg/m^2^)) x TG (mmol/L)/0.81 x 1.52/HDL-C (mmol/L) for females
LAP= [WC (cm)-65] x TG (mmol/L) for males
LAP= [WC (cm)-58]x TG (mmol/L) for females
WHtR = WC (cm)/height (cm)
TyG = Ln ^[TG (mg/dL) x FPG (mg/dL)/2]^
TyG-BMI= TyG x BMI
TyG-WC = TyG x WC (cm)
TyG-WHtR = TyG x WHtR

### 2.4 Covariates

Age, gender, ethnicity (Mexican American, non-Hispanic white, non-Hispanic black and others), family income-poverty ratio (FIPR) level, education level (less than high school, high school or equivalent, and college or above) and other demographic and lifestyle characteristics extracted from household questionnaires and used as covariates. Histories of hypertension, high cholesterol and diabetes referred to self-reported diagnosis of a particular disease. More details of the aforementioned characteristics are publicly available on the NHANES website.

### 2.5 Statistical analysis

The statistical analysis was performed in accordance with the CDC guidelines (https://www.cdc.gov/nchs/nhanes/tutorials/default.aspx), using R software (http://www.R-project.org, The R Foundation R.3.4.3). And MedCalc version 13.0 for Windows (MedCalc Software, Mariakerke, Belgium) was used for significance tests in AUC comparison.

Normally distributed data were expressed as mean ± standard deviation (SD), while abnormally distributed data were expressed as the median of the interquartile range (IQR) (25%, 75%). Characteristics were analyzed between the MAFLD group and the non-MAFLD group using Student’s *t*-test, chi-square test or Mann-Whitney U test, as well as between the NAFLD group and the non-NAFLD group. Three logistic models were employed to estimate the odds ratio (OR) with a 95% confidence interval (CI) for MAFLD/NAFLD using nine indirect indexes (BMI, WC, VAI, LAP, WHtR, TyG, TyG-BMI, TyG-WC and TyG-WHtR) and MAFLD/NAFLD as continuous variables (per inter-quartile range (IQR) increment). Model 1 contained only independent variables. Model 2 was adjusted for gender, age, ethnicity, FIPR and education level. Model 3 was further adjusted for hypertension, high cholesterol and diabetes history. Results were presented with odds ratios (ORs) and confidence intervals (95% CIs). The receiver operating characteristic (ROC) curve and the area under curve (AUC) were used to assess the predictive performance of the nine indexes for MAFLD/NAFLD. DeLong et al’s non-parametric method was used to compare the AUC between TyG-WC and other indexes. The best cutoff values of the nine indexes for predicting MAFLD/NAFLD were determined based on the maximum value of the sum of sensitivity and specificity. Statistical significance was set to *P*<0.05.

## 3 Results

### 3.1 Characteristics of the participants

Of all 5,569 individuals aged ≥20 years in cycle 2017-2018 of NHANES, we excluded those missing important data (**Figure 1**). At last, we included 809 participants with complete ultrasound and required data for the evaluation of MAFLD/NAFLD.

The demographic and clinical characteristics of participants, grouped as non-MAFLD, MAFLD, non-NAFLD and NAFLD were shown in **Table 1**. Among all the 809 participants, there were 478 participants with MAFLD and 499 participants with NAFLD, respectively. The proportion of male and female was 50.43% and 49.57%, respectively. The mean age was 46.0 (33.0, 60.0) years. Participants with and without MAFLD/NAFLD had statistically different baseline characteristics, except for FIPR and education level. However, there was no statistical difference in gender between the two groups with and without MAFLD. Participants with MAFLD/NAFLD were more likely to be older and have hypertension/high cholesterol/diabetes. More importantly, participants with MAFLD/NAFLD had higher BMI/WC/VAI/LAP/WHtR/TyG/TyG-BMI/TyG-WC/TyG-WHtR levels.

**Table 1:** Basic characteristics of participants by MAFLD and NAFLD in NHANES 2017-2018.

Variables	Total (*n*=809)		*p*	Total (*n*=809)		*p*
	non-MAFLD (*n* = 331)	MAFLD (*n* = 478)		non-NAFLD (*n* = 310)	NAFLD (*n* = 499)	
Age	40.00 (28.50, 57.00)	50.00 (37.00, 61.00)	< 0.001	41.00 (29.25, 57.00)	49.00 (36.00, 61.00)	< 0.001
Gender, *n* (%)			0.177			0.032
Female	174 (52.57)	227 (47.49)		169 (54.52)	232 (46.49)	
Male	157 (47.43)	251 (52.51)		141 (45.48)	267 (53.51)	
Ethnicity, *n* (%)			< 0.001			< 0.001
Mexican American	33 (9.97)	77 (16.11)		30 (9.68)	80 (16.03)	
Non-Hispanic Black	105 (31.72)	93 (19.46)		101 (32.58)	97 (19.44)	
Non-Hispanic White	90 (27.19)	164 (34.31)		81 (26.13)	173 (34.67)	
Other	103 (31.12)	144 (30.13)		98 (31.61)	149 (29.86)	
FIPR	2.16 (1.20, 4.27)	2.13 (1.22, 4.13)	0.884	2.20 (1.18, 4.25)	2.11 (1.22, 4.155)	0.880
Education, *n* (%)			0.733			0.654
College or above	195 (58.91)	275 (57.65)		186 (60.00)	284 (57.03)	
High school or equivalent	78 (23.57)	108 (22.64)		70 (22.58)	116 (23.29)	
Less than high school	58 (17.52)	94 (19.71)		54 (17.42)	98 (19.68)	
BMI (kg/m2)	24.00 (21.50, 26.90)	30.80 (27.53, 35.10)	< 0.001	24.15 (21.60, 27.28)	30.30 (27.00, 34.95)	< 0.001
WC	85.50 (78.45, 94.10)	104.40 (95.60, 115.45)	< 0.001	85.70 (78.53, 94.60)	103.70 (94.10, 115.00)	< 0.001
VAI	1.00 (0.67, 1.52)	1.83 (1.27, 2.90)	< 0.001	1.01 (0.67, 1.56)	1.77 (1.23, 2.79)	< 0.001
LAP	21.23 (13.19, 37.72)	58.84 (40.33, 86.40)	< 0.001	21.92 (13.58, 38.86)	57.08 (37.85, 83.82)	< 0.001
WHtR	0.51 (0.47, 0.57)	0.63 (0.57, 0.69)	< 0.001	0.52 (0.47, 0.57)	0.62 (0.57, 0.69)	< 0.001
TyG	8.27 (8.00, 8.62)	8.78 (8.47, 9.18)	< 0.001	8.29 (7.99, 8.64)	8.76 (8.43, 9.16)	< 0.001
TyG-BMI	200.81 (174.19, 227.81)	270.06 (240.02, 314.40)	< 0.001	202.30 (174.18, 231.15)	266.78 (236.94, 312.21)	< 0.001
TyG-WC	707.22 (637.65, 806.62)	927.21 (836.70, 1026.31)	< 0.001	708.82 (639.24, 808.82)	916.18 (824.10, 1019.42)	< 0.001
TyG-WHtR	4.23 (3.83, 4.83)	5.51 (5.00, 6.18)	< 0.001	4.29 (3.84, 4.89)	5.46 (4.94, 6.16)	< 0.001
Hypertension, *n* (%)			< 0.001			< 0.001
no	278 (84.50)	301 (62.97)		258 (83.77)	321 (64.33)	
yes	51 (15.50)	177 (37.03)		50 (16.23)	178 (35.67)	
High cholesterol, *n* (%)			0.002			0.011
no	274 (83.03)	348 (73.42)		255 (82.26)	367 (74.29)	
yes	56 (16.97)	126 (26.58)		55 (17.74)	127 (25.71)	
Diabetes, *n* (%)			< 0.001			< 0.001
no	311 (93.96)	384 (80.34)		290 (93.55)	405 (81.16)	
yes	20 (6.04)	94 (19.66)		20 (6.45)	94 (18.84)	

MAFLD, metabolic dysfunction-associated fatty liver disease; NAFLD, non-alcoholic fatty liver disease; FIPR, family income-poverty ratio; BMI, body mass index; WC, waist circumference; VAI, visceral adiposity index; LAP, lipid accumulation product; WHtR, waist-to-height ratio; TyG, triglyceride and glucose index.

### 3.2 Associations between nine indirect indexes and NAFLD/MAFLD

**Table 2** showed the multi-variate adjusted ORs and 95% CIs of MAFLD/NAFLD risks in relation to the quartile increment of nine indexes levels. After adjusting for gender, age, ethnicity, FIPR and education level, all those nine indexes were positive correlated with the risks of MAFLD/NAFLD. For MAFLD, TyG-WC presented the highest OR (OR = 28.435, 95% CI = 12.121 to 66.705), followed by TyG-WHtR (OR = 26.863, 95% CI = 12.417 to 58.115), TyG-BMI (OR = 17.196, 95% CI = 7.193 to 41.110), LAP (OR = 16.609, 95% CI = 7.927 to 34.797), WC (OR = 15.449, 95% CI = 7.440 to 32.077), WHtR (OR = 15.005, 95% CI = 8.052 to 27.964), BMI (OR = 10.986, 95% CI = 5.317 to 22.698), TyG (OR = 5.901, 95% CI = 3.825 to 9.102), and VAI (OR = 4.651, 95% CI = 2.966 to 7.295). Similar results were found after adjusting for all the covariates.

**Table 2:** Multi-variate adjusted ORs (95% CIs) of NAFLD and MAFLD in relation to quartile increment of nine predictive indexes among participants in NHANES 2017-2018.

Variables	Model 1	*p-Value*	Model 2	*p-Value*	Model 3	*p-Value*
MAFLD						
BMI	9.925[4.677,21.063]	<0.001	10.986[5.317,22.698]	0.001	10.847[5.195,22.650]	0.008
WC	16.011[7.257,35.321]	<0.001	15.449[7.440,32.077]	<0.001	15.638[7.426,32.935]	0.005
VAI	4.000[2.699,5.929]	<0.001	4.651[2.966,7.295]	0.001	4.399[2.698,7.172]	0.010
LAP	15.372[7.152,33.039]	<0.001	16.609[7.927,34.797]	<0.001	15.931[7.720,32.876]	0.005
WHtR	11.323[6.324,20.277]	<0.001	15.005[8.052,27.964]	<0.001	15.399[8.214,28.870]	0.003
TyG	6.178[3.958,9.644]	<0.001	5.901[3.825,9.102]	<0.001	5.768[3.608,9.223]	0.005
TyG-BMI	16.132[6.316,41.202]	<0.001	17.196[7.193,41.110]	0.001	17.118[7.165,40.895]	0.008
TyG-WC	29.436[11.649,74.379]	<0.001	28.435[12.121,66.705]	<0.001	28.877[12.298,67.805]	0.005
TyG-WHtR	19.412[9.256,40.710]	<0.001	26.863[12.417,58.115]	<0.001	27.798[12.960,59.623]	0.003
NAFLD						
BMI	5.775[3.145,10.603]	<0.001	6.047[3.315,11.032]	0.001	5.806[3.106,10.851]	0.012
WC	8.714[4.531,16.759]	<0.001	8.204[4.491,14.985]	<0.001	7.921[4.232,14.826]	0.007
VAI	3.173[2.077,4.846]	<0.001	3.706[2.309,5.948]	0.002	3.557[2.129,5.942]	0.017
LAP	8.779[4.768,16.166]	<0.001	9.731[5.318,17.807]	<0.001	9.350[5.087,17.188]	0.006
WHtR	6.234[3.883,10.008]	<0.001	7.939[4.797,13.140]	<0.001	7.767[4.584,13.160]	0.005
TyG	4.997[3.262,7.656]	<0.001	4.896[3.164,7.577]	<0.001	4.826[2.962,7.861]	0.008
TyG-BMI	8.178[4.005,16.699]	<0.001	8.278[4.199,16.321]	0.001	8.113[4.084,16.116]	0.009
TyG-WC	13.343[6.579,27.061]	<0.001	12.742[6.576,24.689]	<0.001	12.642[6.453,24.766]	0.005
TyG-WHtR	9.329[5.363,16.227]	<0.001	12.202[6.830,21.798]	<0.001	12.283[6.782,22.245]	0.004

MAFLD, metabolic dysfunction-associated fatty liver disease; NAFLD, non-alcoholic fatty liver disease; BMI, body mass index; WC, waist circumference; VAI, visceral adiposity index; LAP, lipid accumulation product; WHtR, waist-to-height ratio; TyG, triglyceride and glucose index. Model 1 included only independent variables; model 2 was additionally adjusted for gender, age, ethnicity, FIPR and education level; and model 3 was further adjusted for the disease history (hypertension, high cholesterol and diabetes).

For NAFLD, TyG-WC presented the highest OR (OR = 12.742, 95% CI = 6.576 to 24.689), followed by TyG-WHtR (OR = 12.202, 95% CI = 6.830 to 21.798), LAP (OR = 9.731, 95% CI = 5.318 to 17.807), TyG-BMI (OR = 8.278, 95% CI = 4.199 to 16.321), WC (OR = 8.204, 95% CI = 4.491 to 14.985), WHtR (OR = 7.939, 95% CI = 4.797 to 13.140), BMI (OR = 6.047, 95% CI = 3.315 to11.032), TyG (OR = 4.896, 95% CI = 3.164 to 7.577), VAI (OR = 3.706, 95% CI = 2.309 to 5.948).

### 3.3 Nine indirect indexes for predicting MAFLD/NAFLD

**Figure 2:**
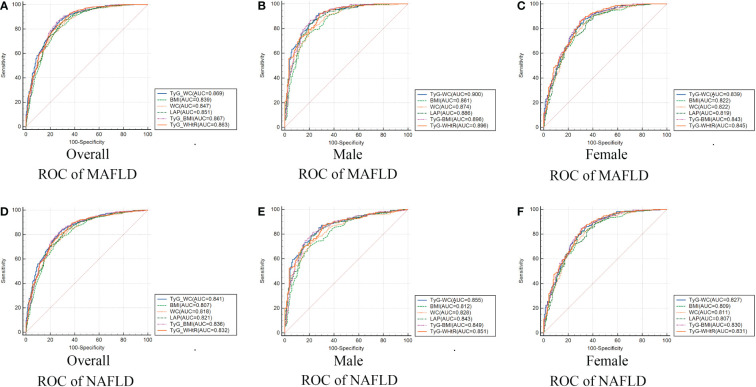
Receiver operating characteristic curves of TyG-WC and other indexes in overall (A/D), male (B/E), female (C/F) for identifying MAFLD/NAFLD. MAFLD, metabolic dysfunction-associated fatty liver disease; NAFLD, non-alcoholic fatty liver disease; TyG, triglyceride and glucose index; WC, waist circumference; BMI, body mass index; LAP, lipid accumulation product; WHtR, waist-to-height ratio.

**Table 3** and **Figure 2** showed the AUC values (95% CI) of the 9 indexes for screening American adults with MAFLD/NAFLD. For MAFLD, TyG-WC presented the highest AUC for male (0.900, 95% CI: 0.867-0.927) and overall (0.869, 95% CI: 0.843-0.891). The optimum cutoff value of TyG-WC was 789.868 (specificity 92.43%, sensitivity 72.61%) for male. However, TyG-WHtR presented the highest AUC for female (0.845, 95% CI: 0.806-0.879), with an optimum cutoff value of 4.821 (specificity: 86.78%, sensitivity: 69.54%). **Table 3** also showed negative predictive value (NPV) and positive predictive value (PPV) of the nine indexes.

**Table 3:** Selected parameters for predicting MAFLD/NAFLD and the corresponding AUC, optimal cut-off values, their sensitivity and specificity, PPV and NPV.

	Gender	Variable	AUC (95%CI)	Cut-off Values	Specificity (%)	Sensitivity (%)	PPV	NPV	*p*-Value
MAFLD	Female	BMI	0.822[0.781,0.859]	26.500	81.94	72.41	0.795	0.754	<0.0001
		WC	0.822[0.781,0.859]	88.600	85.46	66.09	0.767	0.777	<0.0001
		VAI	0.733[0.687,0.776]	1.225	81.5	56.32	0.709	0.700	<0.0001
		LAP	0.819[0.778,0.856]	33.125	86.34	63.79	0.757	0.782	<0.0001
		WHtR	0.833[0.793,0.868]	0.574	84.58	70.69	0.790	0.778	<0.0001
		TyG	0.725[0.679,0.769]	8.535	67.4	70.69	0.750	0.624	<0.0001
		TyG-BMI	0.843[0.804,0.878]	225.138	86.34	70.69	0.794	0.799	<0.0001
		TyG-WC	0.839[0.800,0.874]	755.391	87.67	67.24	0.777	0.807	<0.0001
		TyG-WHtR	0.845[0.806,0.879]	4.821	86.78	69.54	0.788	0.801	<0.0001
	Male	BMI	0.861[0.824,0.893]	27.400	74.5	82.17	0.870	0.668	<0.0001
		WC	0.874[0.838,0.904]	96.300	78.09	79.62	0.860	0.694	<0.0001
		VAI	0.800[0.758,0.838]	1.269	71.71	77.71	0.837	0.632	<0.0001
		LAP	0.886[0.852,0.916]	36.271	83.27	80.89	0.874	0.752	<0.0001
		WHtR	0.870[0.833,0.901]	0.545	82.07	76.43	0.848	0.727	<0.0001
		TyG	0.790[0.748,0.829]	8.527	74.9	72.61	0.814	0.644	<0.0001
		TyG-BMI	0.896[0.863,0.924]	228.023	86.45	79.62	0.871	0.786	<0.0001
		TyG-WC	0.900[0.867,0.927]	789.868	92.43	72.61	0.844	0.857	<0.0001
		TyG-WHtR	0.896[0.862,0.923]	4.476	92.43	71.34	0.838	0.855	<0.0001
	Overall	BMI	0.839[0.811,0.863]	26.700	80.33	74.32	0.819	0.723	<0.0001
		WC	0.847[0.820,0.871]	90.300	87.24	67.07	0.793	0.784	<0.0001
		VAI	0.759[0.728,0.789]	1.186	79.71	62.84	0.756	0.682	<0.0001
		LAP	0.851[0.825,0.875]	33.286	86.61	70.09	0.807	0.784	<0.0001
		WHtR	0.842[0.815,0.866]	0.559	81.38	73.72	0.817	0.733	<0.0001
		TyG	0.758[0.727,0.788]	8.535	71.34	71.6	0.784	0.634	<0.0001
		TyG-BMI	0.867[0.841,0.889]	223.282	88.28	72.21	0.821	0.810	<0.0001
		TyG-WC	0.869[0.843,0.891]	790.927	87.03	72.51	0.821	0.795	<0.0001
		TyG-WHtR	0.863[0.838,0.886]	4.811	82.64	74.62	0.825	0.749	<0.0001
NAFLD	Female	BMI	0.809[0.767,0.846]	26.500	80.17	71.6	0.795	0.725	<0.0001
		WC	0.811[0.769,0.848]	86.400	89.22	59.76	0.753	0.802	<0.0001
		VAI	0.722[0.676,0.766]	1.225	80.6	56.21	0.716	0.679	<0.0001
		LAP	0.807[0.765,0.845]	40.826	71.98	75.74	0.803	0.663	<0.0001
		WHtR	0.820[0.779,0.856]	0.574	82.76	69.82	0.790	0.747	<0.0001
		TyG	0.715[0.668,0.759]	8.535	65.95	69.82	0.750	0.599	<0.0001
		TyG-BMI	0.830[0.789,0.865]	225.138	84.48	69.82	0.794	0.766	<0.0001
		TyG-WC	0.827[0.786,0.863]	755.391	85.78	66.27	0.777	0.772	<0.0001
		TyG-WHtR	0.831[0.791,0.867]	4.821	84.91	68.64	0.788	0.768	<0.0001
	Male	BMI	0.812[0.771,0.849]	27.400	70.04	80.14	0.870	0.586	<0.0001
		WC	0.828[0.787,0.863]	96.300	73.78	78.01	0.864	0.611	<0.0001
		VAI	0.769[0.725,0.809]	1.269	67.79	75.89	0.842	0.554	<0.0001
		LAP	0.843[0.804,0.877]	36.271	78.28	78.72	0.874	0.657	<0.0001
		WHtR	0.823[0.783,0.859]	0.545	77.53	74.47	0.852	0.636	<0.0001
		TyG	0.767[0.722,0.807]	8.512	73.03	70.21	0.823	0.579	<0.0001
		TyG-BMI	0.849[0.811,0.883]	228.023	81.27	77.3	0.871	0.685	<0.0001
		TyG-WC	0.855[0.817,0.888]	831.409	79.03	78.72	0.876	0.665	<0.0001
		TyG-WHtR	0.851[0.813,0.884]	4.476	87.27	68.79	0.841	0.741	<0.0001
	Overall	BMI	0.807[0.779,0.834]	26.700	76.95	72.58	0.819	0.662	<0.0001
		WC	0.818[0.790,0.844]	94.400	74.75	74.52	0.825	0.647	<0.0001
		VAI	0.735[0.703,0.765]	1.186	76.95	61.29	0.762	0.623	<0.0001
		LAP	0.821[0.793,0.847]	33.286	82.97	68.06	0.807	0.713	<0.0001
		WHtR	0.810[0.781,0.836]	0.559	78.16	72.26	0.819	0.673	<0.0001
		TyG	0.741[0.710,0.771]	8.535	68.94	70.65	0.791	0.586	<0.0001
		TyG-BMI	0.836[0.808,0.861]	227.600	81.96	73.23	0.831	0.716	<0.0001
		TyG-WC	0.841[0.814,0.865]	790.927	83.57	70.97	0.823	0.729	<0.0001
		TyG-WHtR	0.832[0.804,0.857]	4.811	79.36	73.23	0.827	0.688	<0.0001

MAFLD, metabolic dysfunction-associated fatty liver disease; NAFLD, non-alcoholic fatty liver disease; BMI, body mass index; WC, waist circumference; VAI, visceral adiposity index; LAP, lipid accumulation product; WHtR, waist-to-height ratio; TyG, triglyceride and glucose index;PPV, positive predictive value; NPV, Negative predictive value.

Similar results were found for NAFLD. TyG-WHtR presented best predictive performance for female, while TyG-WC presented best predictive performance for male (**Table 3**).

### 3.4 Gender difference in AUC values between TyG-WC and other indexes

TyG-WC presented the largest AUC in overall population both with NAFLD and MAFLD. We compared the AUC values between TyG-WC and other eight indexes to explore possible gender differences. **Table 4** showed the gender differences in AUC values between TyG-WC and other indexes for MAFLD/NAFLD. Similar results were found for both MAFLD and NAFLD. For female, the AUC value of TyG-WC was statistically different from that of WC (*P*<0.05), but not statistically different from that of BMI, LAP, TyG-BMI and TyG-WHtR. For male, the AUC value of TyG-WC was statistically different from that of BMI and WC (*P*<0.05), but not statistically different from that of LAP, TyG-BMI and TyG-WHtR. Therefore, TyG-WC, TyG-BMI and TyG-WHtR might have better predictive performance in identifying MAFLD and NAFLD compared to BMI, WC and LAP.

**Table 4:** Gender differences in AUC values between TyG-WC and other indexes.

MAFLD	Difference between Area (95%CI)	*p*-Value	NAFLD	Difference between Area (95%CI)	*p*-Value
Female			Female		
TyG-WC VS BMI	0.017[-0.011,0.045]	0.236	TyG-WC VS BMI	0.018[-0.010,0.046]	0.210
TyG-WC VS WC	0.017[0.002,0.032]	0.028	TyG-WC VS WC	0.016[0.001,0.031]	0.037
TyG-WC VS LAP	0.020[0.000,0.040]	0.051	TyG-WC VS LAP	0.020[0.000,0.040]	0.054
TyG-WC VS TyG-BMI	0.004[-0.016,0.024]	0.686	TyG-WC VS TyG-BMI	0.003[-0.018,0.023]	0.807
TyG-WC VS TyG-WHtR	0.006[-0.004,0.016]	0.258	TyG-WC VS TyG-WHtR	0.004[-0.006,0.015]	0.423
Male			Male		
TyG-WC VS BMI	0.039[0.013,0.064]	0.003	TyG-WC VS BMI	0.043[0.016,0.070]	0.002
TyG-WC VS WC	0.026[0.010,0.042]	0.002	TyG-WC VS WC	0.028[0.011,0.045]	0.001
TyG-WC VS LAP	0.014[-0.004,0.031]	0.126	TyG-WC VS LAP	0.012[-0.006,0.030]	0.193
TyG-WC VS TyG-BMI	0.004[-0.012,0.020]	0.663	TyG-WC VS TyG-BMI	0.006[-0.011,0.023]	0.493
TyG-WC VS TyG-WHtR	0.004[-0.006,0.015]	0.406	TyG-WC VS TyG-WHtR	0.004[-0.007,0.016]	0.429
Overall			Overall		
TyG-WC VS BMI	0.030[0.011,0.049]	0.002	TyG-WC VS BMI	0.033[0.013,0.053]	0.001
TyG-WC VS WC	0.022[0.011,0.033]	0.000	TyG-WC VS WC	0.022[0.011,0.033]	0.000
TyG-WC VS LAP	0.017[0.003,0.032]	0.015	TyG-WC VS LAP	0.019[0.005,0.034]	0.009
TyG-WC VS TyG-BMI	0.002[-0.012,0.016]	0.777	TyG-WC VS TyG-BMI	0.005[-0.009,0.019]	0.502
TyG-WC VS TyG-WHtR	0.005[-0.005,0.016]	0.328	TyG-WC VS TyG-WHtR	0.009[-0.002,0.020]	0.119

MAFLD, metabolic dysfunction-associated fatty liver disease; NAFLD, non-alcoholic fatty liver disease; BMI, body mass index; WC, waist circumference; VAI, visceral adiposity index; LAP, lipid accumulation product; WHtR, waist-to-height ratio; TyG, triglyceride and glucose index.

## 4 Discussion

As the most common chronic liver disease, MAFLD/NAFLD has affected the life of about 1/4 adults worldwide. Increasing studies are exploring easy-to-use, practical, and reliable predictors of MAFLD/NAFLD, which is also one of the urgent needs in clinical practice. Obesity is an independent risk factor of NAFLD. A Meta-analysis showed that the risk of NAFLD in obese individuals was 3.5 times higher than those with normal BMI, and the severity of NAFLD tended to increase in individuals with higher BMI (33). Previous studies suggested that inflammatory mediators such as lipocalin, leptin, and tumor necrosis factor-α secreted by adipocytes (34), especially lipocalin and leptin, could influence the development of NAFLD by regulating hepatic fat accumulation, IR, and fibrosis (35). Obesity-associated IR is considered to be one of the important pathogenic mechanisms of NAFLD (36).BMI, WC, VAI, LAP and WHtR are obesity-associated indexes, TyG is a reliable IR index, and TyG-BMI, TyG-WC and TyG-WHtR are composite indicators combining TyG and anthropometric parameters. Which of the nine indexes will be most closely linked to NAFLD/MAFLD remains to be determined.

We screened nine associated indexes and compared their performance in predicting MAFLD/NAFLD in adults based on previous studies. To the best of our knowledge, this was the first study to investigate the association between indirect indexes (BMI/WC/VAI/LAP/WHtR/TyG/TyG-WC/TyG-BMI/TyG-WHtR) and MAFLD/NAFLD in the U.S. population by different gender groups. Further assessment has been completed to assess the diagnostic utility of nine indexes for MAFLD/NAFLD. We found that all nine indexes were significantly associated with risks of MAFLD/NAFLD. ROC analysis showed that TyG-WC was the best predictor, followed by TyG-WHtR and TyG-BMI, for MAFLD/NAFLD in male participants. TyG-WHtR was the best predictor for MAFLD/NAFLD in female participants. It is notable that these findings highlight the potential impact of gender on the reliability of assessed indexes.

### 4.1 Relationship between BMI/WC/WHtR and MAFLD/NAFLD

BMI is the most commonly used clinical indicator of whole-body adiposity, while WC is more suitable for assessing central obesity. Our findings showed that, in both male and female participants, WC presented significantly better predictive performance for MAFLD/NAFLD than BMI, which suggested that abdominal obesity might be a more accurate and important index of steatosis than overweight measured by BMI. Our findings also showed that it was clinically valuable to include people with normal BMI but abnormal metabolism in the diagnosis of MAFLD.

Previous studies have found that WC may better reflect the risk of obesity-associated diseases compared to BMI (37). Li et al. found that WC was more effective than BMI in predicting metabolic syndrome in patients with T2DM (38). A prospective cohort study of 11,714 participants suggested that increased WC might result in blood pressure elevation even without increase in BMI (39). In addition, Hou et al. found a stronger correlation between WC and diabetes compared to BMI (40). For a specific BMI, a large WC meant two-to three-fold of the risk of developing diabetes and cardiovascular disease (CVD) in the future (41).

Some studies have explored the relationship between WC and MAFLD/NAFLD. Similar to our findings, Motamed et al. (42) found that WC presented excellent performance in the diagnosis of NAFLD(AUC: 0.8533, 95%CI: 0.8419-0.8646)and almost the same predictive power as fatty liver index (FLI), a widely used index for the diagnosis and evaluation of fatty liver development in a number of studies (43, 44).

We found that WC had higher predictive power than BMI, which may be related to the following factors. First, not all patients with NAFLD have an excessive BMI. In all, about 40% people NAFLD worldwide are classified as non-obese and nearly a fifth are lean (45). Second, the distribution of abdominal fat can be used as a marker of ectopic fat in various sites. According to previous study, those with a predominance of abdominal fat and a large WC have more visceral/intra-abdominal fat, expanded (hypertrophic) subcutaneous adipose cells, as well as dysfunctional and inflammatory adipose tissue (45), and thus are more likely to develop metabolic disorders. Third, it is known that unhealthy dietary patterns have a significant role in the development of MAFLD/NAFLD. Interestingly, Ghaemi et al. found that the indirect effect of diet through abdominal circumference was 28 times more than the direct effect on NAFLD and that WC is a powerful mediator in the association between dietary patterns and NAFLD (46), indicating that WC was of great importance in the development of NAFLD.

Notably, WHtR presented the best predictive performance for MAFLD/NAFLD in female participants. A possible explanation is that WHtR is an adjusted indicator with WC and height, so it can better indicate abdominal obesity than WC.

As easy-to-use and cheap indexes, WC and WHtR are important indicators for assessing central obesity and reliable indexes for efficient screening of individuals at high risk of MAFLD/NAFLD.

### 4.2 The relationship between VAI/LAP and MAFLD/NAFLD

As important indexes of visceral adiposity, VAI and LAP had good performance in diagnosing MAFLD/MAFLD in this study, with AUCs of about 0.7 and 0.8, respectively. And elevated VAI and LAP levels were associated with higher risks of MAFLD and NAFLD after adjusting for all covariates, which was consistent with previous findings by Vural and Zhang et al. (47, 48).

Numerous studies have shown the close link between VAI/LAP and metabolic disorders. According to Dong et al. (49), VAI performed better than traditional adiposity index in predicting an unhealthy metabolic phenotype in Chinese children and adolescents (BMI, WC, and WHtR). However, there is a strong correlation between VAI and abnormalities in lipid and glucose levels in obese individuals (50). A 10-year prospective cohort study has shown a link between LAP and incident cardiovascular disease.

Unexpectedly, we found that MAFLD was also strongly correlated with VAI and LAP. Possible explanations could be: First, people with more visceral adipose tissue (VAT) had higher levels of inflammatory cytokines, including C-reactive protein, tumor necrosis factor-α, and interleukin-6, which may cause IR and metabolic problems (51). Second, the enhanced lipolysis in VAT causes an excess of free fatty acids (FFAs) to be released into the portal vein. FFAs with high concentrations can cause IR and intracellular inflammation (52). Third, the elevated FFAs load in NAFLD may impede a β-oxidation, which takes place in the liver mitochondria, leading to the production of reactive oxygen species (53). Oxidative stress as a result causes the initiation and development of fibrosis, inflammation, and liver damage.

It is notable that LAP seems to have a better predictive performance than VAI according to our findings. LAP can be more easily calculated with WC and TG, so it can be widely used in clinical practice.

### 4.3 Relationship between TyG/TyG-BMI/TyG-WC/TyG-WHtR and MAFLD/NAFLD

TyG index has been widely explored in cardiovascular diseases recently. It is considered as a reliable index to predict adverse cardiovascular events and progression of coronary artery calcification in patients with acute coronary syndrome and diabetes (19, 54). Our findings suggested that TyG was strongly linked to NAFLD, with a 4~6-fold increase in MAFLD/NAFLD risk as each quartile increment in TyG. According to ROC analysis, the optimal cut-off point of TyG for MAFLD was 8.535 and the AUC was 0.758 (95% CI 0.727-0.788), which were generally consistent with the previous findings by Zhang et al. (21). Moreover, we further explored the relationship between TyG and MAFLD/NAFLD in different gender subgroups.

We also found that TyG presented lower performance in predicting MAFLD/NAFLD compared to the other eight indexes. TyG is considered as a novel indicator of IR, but previous study found that a significant number patients with fatty liver remained insulin sensitive and 37% of these patients presented no metabolic syndrome, prediabetes or diabetes (55). We therefore speculate that this may be a reason why the TyG has lower predictive performance than other indexes.

Interestingly, we found that TyG-BMI, TyG-WC and TyG-WHtR had higher predictive performance for MAFLD/NAFLD in overall population, which was similar to the findings by Sheng et al. (56). A possible explanation could be that TyG-BMI, TyG-WC and TyG-BMI were adjusted indexes with indicators of insulin sensitivity and obesity including glucose, insulin level, BMI, WC and WHtR, which were also suggested with good predictive performance for T2DM in previous study (57). Our findings further confirmed the significant contribution of obesity and IR in the development of MAFLD/NAFLD.

### 4.4 Strengths and limitations

Strengths: For the first time, we explored the differences in the performance of nine indexes in predicting both MAFLD and NAFLD, providing a reliable reference for efficient and accurate screening in clinical settings. Furthermore, there are few related clinical studies on MAFLD, so our findings contribute some evidence to the scant research. Last, the study findings were based on the high-quality anthropometric and laboratory data from the NHANES database, which was comprehensive and representative of the population on a national level.

Limitations: First, rather than using the gold standard in histology, the diagnosis of hepatic steatosis was based on imaging (FibroScan). Second, despite the fact that we adjusted multiple covariates in the study, there may still be other potential confounders, such as physical activity and food intakes. Third, in this cross-sectional study, we were unable to confirm a cause-effect relationship between the risk of MAFLD/NAFLD and the 9 indexes (BMI, WC, VAI, LAP, WHtR, TyG, TyG-WC, TyG-BMI and TyG-WHtR). More large-scale and prospective cohort studies should be encouraged in the future.

## 5 Conclusion

Our study suggests that BMI/WC/VAI/LAP/WHtR/TyG/TyG-WC/TyG-BMI/TyG-WHtR are important reference indexes for identifying the risks of MAFLD/NAFLD. TyG-WC presents the best predictive performance in male, while TyG-WHtR presents the best predictive performance in female. Further prospective studies are needed before definite conclusions about the best predictor of MAFLD/NAFLD can be made.

## Data availability statement

Publicly available datasets were analyzed in this study. This data can be found here: Centers for disease control and prevention. 2017: National Health and Nutrition Examination Survey (NHANES). U.S. Department of health and human services. Available from https://wwwn.cdc.gov/nchs/data/nhanes/2017-2018/manuals/2017_MEC_Laboratory_Procedures_Manual.pdf accessed 31 March 2020.

## Ethics statement

The studies involving human participants were reviewed and approved by The Research Ethics Review Board of the National Center for Health Statistics examined and approved the NHANES protocol. Before taking part, each participant completed a written statement of informed consent. The patients/participants provided their written informed consent to participate in this study.

## Author contributions

Conceptualization, HP and WL. Methodology, HP, LP and SR. Formal analysis, MW, SH and MZ. Data curation, ZC, ZY, LX and QY. Writing—original draft preparation, HP, LP and SR. Writing—review and editing, WL. Visualization, MW, SH and MZ. All authors contributed to the article and approved the submitted version.

## References

[1] Younossi ZM Koenig AB Abdelatif D Fazel Y Henry L Wymer M. Global epidemiology of nonalcoholic fatty liver disease-Meta-Analytic assessment of prevalence, incidence, and outcomes. Hepatol (Baltimore Md) (2016) 64(1):73–84. doi: 10.1002/hep.28431 26707365

[2] Targher G Corey KE Byrne CD Roden M. The complex link between nafld and type 2 diabetes mellitus - mechanisms and treatments. Nat Rev Gastroenterol Hepatol (2021) 18(9):599–612. doi: 10.1038/s41575-021-00448-y 33972770

[3] Zhao YC Zhao GJ Chen Z She ZG Cai J Li H. Nonalcoholic fatty liver disease: An emerging driver of hypertension. Hypertension (Dallas Tex: 1979) (2020) 75(2):275–84. doi: 10.1161/hypertensionaha.119.13419 31865799

[4] Eslam M Sanyal AJ George J. Mafld: A consensus-driven proposed nomenclature for metabolic associated fatty liver disease. Gastroenterology (2020) 158(7):1999–2014.e1. doi: 10.1053/j.gastro.2019.11.312 32044314

[5] Lin S Huang J Wang M Kumar R Liu Y Liu S. Comparison of mafld and nafld diagnostic criteria in real world. Liver Int (2020) 40(9):2082–9. doi: 10.1111/liv.14548 32478487

[6] Targher G Tilg H Byrne CD. Non-alcoholic fatty liver disease: A multisystem disease requiring a multidisciplinary and holistic approach. Lancet Gastroenterol Hepatol (2021) 6(7):578–88. doi: 10.1016/s2468-1253(21)00020-0 33961787

[7] Younossi ZM Blissett D Blissett R Henry L Stepanova M Younossi Y. The economic and clinical burden of nonalcoholic fatty liver disease in the united states and Europe. Hepatol (Baltimore Md) (2016) 64(5):1577–86. doi: 10.1002/hep.28785 27543837

[8] Estes C Razavi H Loomba R Younossi Z Sanyal AJ. Modeling the epidemic of nonalcoholic fatty liver disease demonstrates an exponential increase in burden of disease. Hepatol (Baltimore Md) (2018) 67(1):123–33. doi: 10.1002/hep.29466 PMC5767767 28802062

[9] Castera L Friedrich-Rust M Loomba R. Noninvasive assessment of liver disease in patients with nonalcoholic fatty liver disease. Gastroenterology (2019) 156(5):1264–81.e4. doi: 10.1053/j.gastro.2018.12.036 30660725 PMC7505052

[10] Yu Q Huang S Xu TT Wang YC Ju S. Measuring brown fat using mri and implications in the metabolic syndrome. J Magnetic Resonance Imaging (2021) 54(5):1377–92. doi: 10.1002/jmri.27340 33047448

[11] Staiano AE Gupta AK Katzmarzyk PT. Cardiometabolic risk factors and fat distribution in children and adolescents. J Pediatr (2014) 164(3):560–5. doi: 10.1016/j.jpeds.2013.10.064 PMC3943888 24315509

[12] Rotar O Boyarinova M Orlov A Solntsev V Zhernakova Y Shalnova S. Metabolically healthy obese and metabolically unhealthy non-obese phenotypes in a Russian population. Eur J Epidemiol (2017) 32(3):251–4. doi: 10.1007/s10654-016-0221-z 28039558

[13] Blüher M. Metabolically healthy obesity. Endocrine Rev (2020) 41(3):bnaa004. doi: 10.1210/endrev/bnaa004 32128581 PMC7098708

[14] Amato MC Giordano C Galia M Criscimanna A Vitabile S Midiri M. Visceral adiposity index: A reliable indicator of visceral fat function associated with cardiometabolic risk. Diabetes Care (2010) 33(4):920–2. doi: 10.2337/dc09-1825 PMC2845052 20067971

[15] Xu C Ma Z Wang Y Liu X Tao L Zheng D. Visceral adiposity index as a predictor of nafld: A prospective study with 4-year follow-up. Liver International: Off J Int Assoc Study Liver (2018) 38(12):2294–300. doi: 10.1111/liv.13941 30099825

[16] Kahn HS. The “Lipid accumulation product” performs better than the body mass index for recognizing cardiovascular risk: A population-based comparison. BMC Cardiovasc Disord (2005) 5:26. doi: 10.1186/1471-2261-5-26 16150143 PMC1236917

[17] Dai H Wang W Chen R Chen Z Lu Y Yuan H. Lipid accumulation product is a powerful tool to predict non-alcoholic fatty liver disease in Chinese adults. Nutr Metab (2017) 14:49. doi: 10.1186/s12986-017-0206-2 PMC5539973 28775758

[18] Alizargar J Bai CH Hsieh NC Wu SV. Use of the triglyceride-glucose index (Tyg) in cardiovascular disease patients. Cardiovasc Diabetol (2020) 19(1):8. doi: 10.1186/s12933-019-0982-2 31941513 PMC6963998

[19] Wang L Cong HL Zhang JX Hu YC Wei A Zhang YY. Triglyceride-glucose index predicts adverse cardiovascular events in patients with diabetes and acute coronary syndrome. Cardiovasc Diabetol (2020) 19(1):80. doi: 10.1186/s12933-020-01054-z 32534586 PMC7293784

[20] Lv L Zhou Y Chen X Gong L Wu J Luo W. Relationship between the tyg index and diabetic kidney disease in patients with type-2 diabetes mellitus. Diabetes Metab Syndrome Obes (2021) 14:3299–306. doi: 10.2147/dmso.S318255 PMC8296712 34305401

[21] Zhang S Du T Zhang J Lu H Lin X Xie J. The triglyceride and glucose index (Tyg) is an effective biomarker to identify nonalcoholic fatty liver disease. Lipids Health Dis (2017) 16(1):15. doi: 10.1186/s12944-017-0409-6 28103934 PMC5248473

[22] Tutunchi H Naeini F Mobasseri M Ostadrahimi A. Triglyceride glucose (Tyg) index and the progression of liver fibrosis: A cross-sectional study. Clin Nutr ESPEN (2021) 44:483–7. doi: 10.1016/j.clnesp.2021.04.025 34330512

[23] Song S Son DH Baik SJ Cho WJ Lee YJ. Triglyceride glucose-waist circumference (Tyg-wc) is a reliable marker to predict non-alcoholic fatty liver disease. Biomedicines (2022) 10(9):2251. doi: 10.3390/biomedicines10092251 36140352 PMC9496145

[24] Hu H Han Y Cao C He Y. The triglyceride glucose-body mass index: A non-invasive index that identifies non-alcoholic fatty liver disease in the general Japanese population. J Trans Med (2022) 20(1):398. doi: 10.1186/s12967-022-03611-4 PMC9446832 36064712

[25] Malek M Khamseh ME Chehrehgosha H Nobarani S Alaei-Shahmiri F. Triglyceride glucose-waist to height ratio: A novel and effective marker for identifying hepatic steatosis in individuals with type 2 diabetes mellitus. Endocrine (2021) 74(3):538–45. doi: 10.1007/s12020-021-02815-w 34355342

[26] Wolffenbuttel BHR Heiner-Fokkema MR Green R Gans ROB. Relationship between serum B12 concentrations and mortality: Experience in nhanes. BMC Med (2020) 18(1):307. doi: 10.1186/s12916-020-01771-y 33032595 PMC7545540

[27] Eslam M Newsome PN Sarin SK Anstee QM Targher G Romero-Gomez M. A new definition for metabolic dysfunction-associated fatty liver disease: An international expert consensus statement. J Hepatol (2020) 73(1):202–9. doi: 10.1016/j.jhep.2020.03.039 32278004

[28] Mikolasevic I Orlic L Franjic N Hauser G Stimac D Milic S. Transient elastography (Fibroscan(^®^)) with controlled attenuation parameter in the assessment of liver steatosis and fibrosis in patients with nonalcoholic fatty liver disease - where do we stand? World J Gastroenterol (2016) 22(32):7236–51. doi: 10.3748/wjg.v22.i32.7236 PMC4997649 27621571

[29] Liao Y Wu N Wang K Wang M Wang Y Gao J. Otub1 promotes progression and proliferation of prostate cancer *Via* deubiquitinating and stabling cyclin E1. Front Cell Dev Biol (2020) 8:617758. doi: 10.3389/fcell.2020.617758 33537306 PMC7848094

[30] Bawadi H Abouwatfa M Alsaeed S Kerkadi A Shi Z. Body shape index is a stronger predictor of diabetes. Nutrients (2019) 11(5):1018. doi: 10.3390/nu11051018 31067681 PMC6566958

[31] Kuznik A Mardekian J. Trends in utilization of lipid- and blood pressure-lowering agents and goal attainment among the U.S. diabetic population, 1999-2008. Cardiovascular diabetology (2011) 10:31. doi: 10.1186/1475-2840-10-31 21496321 PMC3098774

[32] Simental-Mendía LE Rodríguez-Morán M Guerrero-Romero F. The product of fasting glucose and triglycerides as surrogate for identifying insulin resistance in apparently healthy subjects. Metab Syndrome Related Disord (2008) 6(4):299–304. doi: 10.1089/met.2008.0034 19067533

[33] Li L Liu DW Yan HY Wang ZY Zhao SH Wang B. Obesity is an independent risk factor for non-alcoholic fatty liver disease: Evidence from a meta-analysis of 21 cohort studies. Obes Rev (2016) 17(6):510–9. doi: 10.1111/obr.12407 27020692

[34] Henao-Mejia J Elinav E Jin C Hao L Mehal WZ Strowig T. Inflammasome-mediated dysbiosis regulates progression of nafld and obesity. Nature (2012) 482(7384):179–85. doi: 10.1038/nature10809 PMC3276682 22297845

[35] Boutari C Mantzoros CS. Adiponectin and leptin in the diagnosis and therapy of nafld. Metabolism (2020) 103:154028. doi: 10.1016/j.metabol.2019.154028 31785257

[36] Watt MJ Miotto PM De Nardo W Montgomery MK. The liver as an endocrine organ-linking nafld and insulin resistance. Endocrine Rev (2019) 40(5):1367–93. doi: 10.1210/er.2019-00034 31098621

[37] Müller MJ Lagerpusch M Enderle J Schautz B Heller M Bosy-Westphal A. Beyond the body mass index: Tracking body composition in the pathogenesis of obesity and the metabolic syndrome. Obes Rev (2012) 13 Suppl 2:6–13. doi: 10.1111/j.1467-789X.2012.01033.x 23107255

[38] Li H Wang Q Ke J Lin W Luo Y Yao J. Optimal obesity- and lipid-related indices for predicting metabolic syndrome in chronic kidney disease patients with and without type 2 diabetes mellitus in China. Nutrients (2022) 14(7):1334. doi: 10.3390/nu14071334 35405947 PMC9002364

[39] Wang Y Howard AG Adair LS Wang H Avery CL Gordon-Larsen P. Waist circumference change is associated with blood pressure change independent of bmi change. Obes (Silver Spring Md) (2020) 28(1):146–53. doi: 10.1002/oby.22638 PMC6925347 31755247

[40] Hou X Chen S Hu G Chen P Wu J Ma X. Stronger associations of waist circumference and waist-to-Height ratio with diabetes than bmi in Chinese adults. Diabetes Res Clin Pract (2019) 147:9–18. doi: 10.1016/j.diabres.2018.07.029 30144478

[41] Després JP Lemieux I. Abdominal obesity and metabolic syndrome. Nature (2006) 444(7121):881–7. doi: 10.1038/nature05488 17167477

[42] Motamed N Sohrabi M Ajdarkosh H Hemmasi G Maadi M Sayeedian FS. Fatty liver index vs waist circumference for predicting non-alcoholic fatty liver disease. World J Gastroenterol (2016) 22(10):3023–30. doi: 10.3748/wjg.v22.i10.3023 PMC4779925 26973398

[43] Li L Huang Q Yang L Zhang R Gao L Han X. The association between non-alcoholic fatty liver disease (Nafld) and advanced fibrosis with serological vitamin B12 markers: Results from the nhanes 1999-2004. Nutrients (2022) 14(6):1224. doi: 10.3390/nu14061224 35334881 PMC8948655

[44] Tan SY Georgousopoulou EN Cardoso BR Daly RM George ES. Associations between nut intake, cognitive function and non-alcoholic fatty liver disease (Nafld) in older adults in the united states: Nhanes 2011-14. BMC Geriatrics (2021) 21(1):313. doi: 10.1186/s12877-021-02239-1 34001034 PMC8127249

[45] Ye Q Zou B Yeo YH Li J Huang DQ Wu Y. Global prevalence, incidence, and outcomes of non-obese or lean non-alcoholic fatty liver disease: A systematic review and meta-analysis. Lancet Gastroenterol Hepatol (2020) 5(8):739–52. doi: 10.1016/s2468-1253(20)30077-7 32413340

[46] Ghaemi A Hosseini N Osati S Naghizadeh MM Dehghan A Ehrampoush E. Waist circumference is a mediator of dietary pattern in non-alcoholic fatty liver disease. Sci Rep (2018) 8(1):4788. doi: 10.1038/s41598-018-23192-x 29555959 PMC5859081

[47] Vural Keskinler M Mutlu HH Sirin A Erkalma Senates B Colak Y Tuncer I. Visceral adiposity index as a practical tool in patients with biopsy-proven nonalcoholic fatty liver Disease/Nonalcoholic steatohepatitis. Metab Syndrome Related Disord (2021) 19(1):26–31. doi: 10.1089/met.2020.0054 32898457

[48] Zhang Y Li B Liu N Wang P He J. Evaluation of different anthropometric indicators for screening for nonalcoholic fatty liver disease in elderly individuals. Int J Endocrinol (2021) 2021:6678755. doi: 10.1155/2021/6678755 33574841 PMC7861948

[49] Dong Y Bai L Cai R Zhou J Ding W. Visceral adiposity index performed better than traditional adiposity indicators in predicting unhealthy metabolic phenotype among Chinese children and adolescents. Sci Rep (2021) 11(1):23850. doi: 10.1038/s41598-021-03311-x 34903825 PMC8668984

[50] Jabłonowska-Lietz B Wrzosek M Włodarczyk M Nowicka G. New indexes of body fat distribution, visceral adiposity index, body adiposity index, waist-to-Height ratio, and metabolic disturbances in the obese. Kardiol Polska (2017) 75(11):1185–91. doi: 10.5603/KP.a2017.0149 28715064

[51] Fontana L Eagon JC Trujillo ME Scherer PE Klein S. Visceral fat adipokine secretion is associated with systemic inflammation in obese humans. Diabetes (2007) 56(4):1010–3. doi: 10.2337/db06-1656 17287468

[52] Stefan N Kantartzis K Häring HU. Causes and metabolic consequences of fatty liver. Endocrine Rev (2008) 29(7):939–60. doi: 10.1210/er.2008-0009 18723451

[53] Boden G She P Mozzoli M Cheung P Gumireddy K Reddy P. Free fatty acids produce insulin resistance and activate the proinflammatory nuclear factor-kappab pathway in rat liver. Diabetes (2005) 54(12):3458–65. doi: 10.2337/diabetes.54.12.3458 16306362

[54] Park K Ahn CW Lee SB Kang S Nam JS Lee BK. Elevated tyg index predicts progression of coronary artery calcification. Diabetes Care (2019) 42(8):1569–73. doi: 10.2337/dc18-1920 31182490

[55] Stefan N Häring HU. The metabolically benign and malignant fatty liver. Diabetes (2011) 60(8):2011–7. doi: 10.2337/db11-0231 PMC3142070 21788578

[56] Sheng G Lu S Xie Q Peng N Kuang M Zou Y. The usefulness of obesity and lipid-related indices to predict the presence of non-alcoholic fatty liver disease. Lipids Health Dis (2021) 20(1):134. doi: 10.1186/s12944-021-01561-2 34629059 PMC8502416

[57] Er LK Wu S Chou HH Hsu LA Teng MS Sun YC. Triglyceride glucose-body mass index is a simple and clinically useful surrogate marker for insulin resistance in nondiabetic individuals. PloS One (2016) 11(3):e0149731. doi: 10.1371/journal.pone.0149731 26930652 PMC4773118

